# Universal Dried Blood Spot Screening for Congenital Cytomegalovirus: A Slovenian National Implementation Pilot

**DOI:** 10.3390/ijns12030048

**Published:** 2026-06-29

**Authors:** Nika Eržen, Jernej Kovač, Barbka Repič Lampret, Urh Grošelj, Aneta Soltirovska Šalamon, Gregor Nosan

**Affiliations:** 1 Department of Neonatology, University Children’s Hospital, University Medical Centre Ljubljana, Bohoričeva 20, 1000 Ljubljana, Slovenia; nika.erzen@kclj.si (N.E.); aneta.soltirovska@kclj.si (A.S.Š.); 2 Faculty of Medicine, University of Ljubljana, Vrazov trg 2, 1000 Ljubljana, Slovenia; jernej.kovac@kclj.si (J.K.); urh.groselj@kclj.si (U.G.); 3 Clinical Institute of Special Laboratory Diagnostics, University Medical Centre Ljubljana, Vrazov trg 1, 1000 Ljubljana, Slovenia; barbka.repic@kclj.si; 4 Department of Endocrinology, Diabetes and Metabolic Diseases, University Children’s Hospital, University Medical Centre Ljubljana, Bohoričeva 20, 1000 Ljubljana, Slovenia; 5 Centre for Rare Diseases, University Children’s Hospital, University Medical Centre Ljubljana, Bohoričeva 20, 1000 Ljubljana, Slovenia

**Keywords:** cytomegalovirus, CMV, congenital cytomegalovirus infection, newborn screening, NBS, universal screening, dried blood spots, DBS, public health policy

## Abstract

Congenital cytomegalovirus infection (cCMV) is the most common congenital infection and an important cause of sensorineural hearing loss and neurodevelopmental impairment, yet many affected infants remain undetected under selective screening approaches. We conducted a prospective national pilot study to evaluate the feasibility and diagnostic yield of universal dried blood spot (DBS)-based screening for cCMV within the Slovenian newborn screening program. DBS samples collected within 72 h of life were tested by polymerase chain reaction (PCR), and screen-positive newborns underwent confirmatory urine PCR within 21 days together with standardized clinical evaluation. Among 5556 screened newborns, 13 (0.23%) screened positive and cCMV was confirmed in 10, corresponding to a lower-bound birth prevalence of 1.80 per 1000 live births (95% confidence interval, 0.98–3.31), because confirmatory testing was limited to DBS-positive newborns. None of the confirmed cases were clinically suspected at birth, and all passed newborn hearing screening. Six infants met protocol-defined criteria for symptomatic cCMV and received valganciclovir. Historical registry-based clinical case ascertainment in Slovenia corresponded to 0.09 detected cases per 1000 live births. These findings demonstrate the feasibility of universal DBS-based cCMV screening within an established newborn screening infrastructure and suggest substantial under-ascertainment under selective clinical detection pathways.

## 1. Introduction

Congenital cytomegalovirus infection (cCMV) is the most common congenital infection worldwide and an important cause of sensorineural hearing loss (SNHL) and neurodevelopmental impairment in children. The pooled global birth prevalence has been estimated to be 0.67% (95% confidence interval (CI), 0.54–0.83%), with generally lower rates reported in high-income settings [1]. In Slovenia, a previous urine-based newborn screening (NBS) study reported a birth prevalence of 0.14% (95% CI, 0.05–0.39%) [2], identifying four cases among 2841 newborns. The diagnosis of cCMV was confirmed in all four PCR-positive newborns by viral isolation from urine and detection of cytomegalovirus (CMV) deoxyribonucleic acid (DNA) in umbilical cord blood collected immediately after birth. CMV DNA was also detected in dried blood spot (DBS) samples in two of the four infants. This study was designed as an epidemiological investigation rather than a population-based screening program.

Despite measurable burden of cCMV, population-level identification of affected infants remains limited. Most countries, including Slovenia, rely on selective screening restricted to newborns with suggestive clinical signs or failed hearing screening [3,4]. This approach has important limitations because most infants with cCMV are asymptomatic at birth and pass newborn hearing screening [5]. As a result, many cases are not recognized during the neonatal period, delaying etiological diagnosis, structured follow-up, and consideration of antiviral therapy where clinically indicated [6].

Universal screening has therefore attracted increasing interest, but its broader implementation remains debated. Key concerns include diagnostic performance, cost-effectiveness, and the clinical implications of identifying infants who may remain asymptomatic [6,7,8,9]. Detection of CMV DNA in urine within the first 21 days of life remains the diagnostic gold standard [9,10,11]. However, DBSs, which are already collected routinely within established newborn screening (NBS) programs, represent an attractive screening specimen because they allow testing to be integrated into existing laboratory and logistical pathways without additional sampling. Although DBS-based assays have lower analytical sensitivity than urine- or saliva-based approaches [12,13,14,15,16,17], their scalability and programmatic feasibility make them particularly relevant for population-based screening strategies. Large-scale programs in Minnesota, Ontario and New York have demonstrated that DBS-based cCMV screening can be incorporated into established NBS infrastructures [18,19,20].

Against this background, the primary aim of this national pilot study was to evaluate the feasibility of integrating universal DBS screening for cCMV into the existing Slovenian newborn screening infrastructure and to assess its diagnostic yield by comparison with historical registry-based clinical case ascertainment.

## 2. Materials and Methods

### 2.1. Study Design and Population

This prospective national pilot study included newborns delivered in all Slovenian maternity hospitals between May and September 2023. DBS samples were collected as part of the routine national NBS program within the first 48–72 h of life [21]. For newborns born at home, DBS samples were collected during the first postnatal visit to the maternity hospital; therefore, home births were not systematically excluded from the screening pathway.

### 2.2. Specimen Collection and Screening Procedure

DBS samples were collected on standardized filter paper cards in accordance with the national NBS protocol. For initial CMV DNA testing, one 3.2 mm punch per newborn was used. DNA was extracted from DBSs using the alkaline-based Eonis DNA Extraction Kit (Revvity, Inc., Waltham, MA, USA). A single 3.2 mm DBS punch was placed into an individual well of a 96-well plate, hydrated with 50 µL of Eonis DNA Wash Buffer, and incubated at room temperature for 8 min on a shaker at 700 rpm. The supernatant was then removed and replaced with 50 µL of Eonis DNA Elution Buffer, followed by incubation at 70 °C for 30 min with shaking at 700 rpm to release DNA from the DBS. Subsequently, 8 µL of internal control (IC) was added to each sample. For downstream CMV detection, 3.5 µL of the extracted DNA was used in a (semi)quantitative polymerase chain reaction (qPCR). CMV DNA was detected using a GeneProof^®^ Cytomegalovirus PCR Kit (GeneProof, Brno, Czech Republic) with minor protocol modifications: 3.5 µL of sample was added to 10 µL of GeneProof Master Mix in a 0.1 mL 96-well qPCR plate (MicroAmp™ Fast Optical 96-Well Reaction Plate; Thermo Fisher Scientific, Waltham, MA, USA). Plates were sealed and run on a qPCR instrument according to the manufacturer’s instructions. Samples with a cycle threshold (Ct) value < 40 for the CMV target and successful IC amplification were considered positive. Samples with no detectable CMV signal and successful IC amplification were considered negative. All initially negative samples were re-tested once using a new 3.2 mm punch from the original DBS card. If IC amplification failed and no CMV signal was detected, the analysis was repeated using a new 3.2 mm punch from the original DBS card. All initially positive samples were re-tested in triplicate using newly punched 3.2 mm disks from the original DBS card. A screening result was considered positive if at least one of the three replicate reactions yielded a positive result. Laboratory personnel were blinded to all clinical data.

### 2.3. Confirmatory Testing and Clinical Evaluation

Newborns with a positive DBS screening result were referred for confirmatory testing. cCMV was confirmed by detection of CMV DNA in urine collected within the first 21 days of life, in accordance with international consensus recommendations [9]. Urine CMV quantitative PCR testing was performed by the national reference laboratory for CMV infections. A negative confirmatory urine PCR result ruled out cCMV, whereas a positive result confirmed the diagnosis. All newborns diagnosed with cCMV underwent a comprehensive postnatal assessment as outlined in the national protocol [4]. This standardized evaluation included a thorough physical examination, laboratory investigations (complete blood count, serum electrolytes, urea, creatinine, liver enzymes, total and direct bilirubin), neuroimaging, abdominal ultrasonography (US), and audiological and ophthalmological evaluations. Neuroimaging involved brain US, performed by neonatologists specialized in neonatal imaging, and, when necessary, brain magnetic resonance imaging (MRI). Brain US was performed using a Canon Toshiba Xario^®^ 200 ultrasound system (Canon Medical Systems Corporation, Otawara, Japan) with a multifrequency transducer (5–8 MHz) to optimize spatial resolution. Brain MRI was performed on a 1.5-Tesla or 3-Tesla whole-body scanner (Siemens Healthineers, Erlangen, Germany) using a dedicated head coil. The standard MRI protocol included sagittal T1-weighted images, axial T1- and T2-weighted images, diffusion-weighted imaging (DWI) with apparent diffusion coefficient (ADC) mapping, and susceptibility-weighted imaging (SWI). Diffusion-weighted MRI was performed using b-values of 1600 s/mm^2^ at 3T and 1000 s/mm^2^ at 1.5T. Brain MRI findings were independently evaluated by experienced pediatric neuroradiologists. Abdominal US was performed using a Canon Toshiba Xario^®^ 200 ultrasound system (Canon Medical Systems Corporation, Otawara, Japan) with a broadband curved array (convex) transducer (1–6 MHz). Audiological assessment included automated auditory brainstem response (AABR) testing performed using an ALGO^®^ 7i Newborn Hearing Screener (Natus Medical Incorporated, Pleasanton, CA, USA). Screening was conducted according to the manufacturer’s automated pass/refer criteria using click stimuli at a standard screening intensity of 35 dB normalized hearing level (nHL). Although brainstem evoked response audiometry (BERA) and/or auditory steady-state response (ASSR) testing is part of the national diagnostic protocol, BERA equipment was temporarily unavailable during the study period. Therefore, initial hearing assessment relied on AABR screening, with comprehensive audiological evaluation performed during follow-up. Visual assessment was performed by pediatric ophthalmologists. Asymptomatic cCMV was defined as confirmed cCMV in the absence of abnormalities on physical examination, laboratory investigations, neuroimaging, abdominal US, ophthalmologic examination, and hearing assessment. Symptomatic cCMV was defined as confirmed cCMV with one or more clinical, laboratory, neuroimaging, abdominal ultrasound, ophthalmological, or audiological findings attributable to cCMV, in accordance with national recommendations and international guidelines [4,9]. A detailed description of the national protocol for the postnatal assessment and follow-up of infants with cCMV is provided in the Supplementary Materials.

### 2.4. Criteria for Antiviral Treatment

Antiviral treatment with oral valganciclovir, the treatment of choice for cCMV, was offered to newborns with confirmed cCMV who met predefined national treatment criteria, in accordance with international consensus recommendations and contemporary clinical guidelines available and in use during the study period [4,9,12,22]. Oral valganciclovir, where indicated, was administered at a dose of 16 mg/kg every 12 h, initiated as soon as possible after confirmation of infection, preferably within the first month of life, and continued for a total duration of six months in infants with central nervous system involvement or multisystem disease. If selected cases with isolated hepatitis or isolated thrombocytopenia were present, a shorter treatment duration of at least six weeks was considered, with extension based on clinical and laboratory response. Intravenous ganciclovir was reserved for infants unable to tolerate enteral therapy or those presenting with severe or life-threatening disease [9]. Eligibility for antiviral treatment required a gestational age of ≥32 weeks and the presence of at least one of the following: central nervous system involvement, moderate or severe organ involvement, life-threatening disease, isolated SNHL, or chorioretinitis [4,9,22]. Treatment decisions were made by consensus among neonatologists with expertise in cCMV. Borderline or indeterminate findings were reviewed within a multidisciplinary team involving pediatric neurology, neuroradiology, and pediatric infectious disease specialists. Antiviral therapy was initiated following detailed parental counseling and informed consent. Regular laboratory monitoring was performed because of potential drug-related adverse effects and included a complete blood count, liver enzymes, urea, creatinine, and serum electrolytes. Neutropenia was defined as an absolute neutrophil count < 1000/mm^3^; treatment was temporarily discontinued if the neutrophil count fell below 500/mm^3^ [9]. Additional monitored adverse effects included anemia, thrombocytopenia, hepatotoxicity, renal impairment and gastrointestinal symptoms, including diarrhea and nausea. Laboratory assessments were performed weekly during the first 4–6 weeks of treatment and monthly thereafter until completion of therapy [4,9].

### 2.5. Data Collection and Analysis

Clinical, laboratory, and imaging data were extracted from hospital electronic medical records using standardized procedures. Historical registry-based data on clinically identified cCMV cases and annual live-birth data in Slovenia for 2003–2022 were obtained from the Perinatal Information System of the Republic of Slovenia [23]. Categorical variables were presented as counts and percentages, and continuous variables as medians with interquartile ranges (IQR). The lower-bound birth prevalence in the screened cohort was defined as the number of urine-confirmed cCMV cases per 1000 live births during the study period. Ninety-five percent CIs were calculated using the Wilson method. Detection rates between the screened cohort and the historical clinically ascertained comparison cohort were compared using Fisher’s exact test. All tests were two-sided, with *p* < 0.05 considered statistically significant. This study was designed as a feasibility and implementation pilot rather than a diagnostic accuracy validation study.

### 2.6. Ethical Approval

The study was approved by the Slovenian National Medical Ethics Committee (reference number 0120-38/2023/4). Primary screening was conducted within the framework of the national NBS program. Newborns with a positive screening result were invited for clinical evaluation. Written informed consent was obtained from parents for confirmatory diagnostic testing and for ongoing clinical evaluation and follow-up.

## 3. Results

### 3.1. Screening Outcomes

During the study period, a total of 5558 newborns were eligible for screening. DBS screening was performed in 5556 newborns, while parents of two newborns declined participation. Among 5556 screened newborns, 13 (0.23%) had positive DBS screening results. The study period (May–September 2023) corresponded to approximately one-third of all live births in Slovenia in 2023 [23]. Confirmatory urine PCR identified cCMV in 10 newborns, while three had false-positive DBS results, corresponding to a false-positive rate of 0.54 per 1000 screened newborns. The positive predictive value of DBS screening in this pilot was 76.9% (10/13). Based on the 10 confirmed cases, the estimated lower-bound birth prevalence was 1.80 per 1000 live births (95% CI, 0.98–3.31). Because confirmatory testing was restricted to DBS-positive newborns, potential false-negative DBS results could not be identified, and sensitivity, specificity, and negative predictive value could not be formally estimated within this pilot design. No cases of clinically suspected cCMV following a negative DBS screening result were observed during the study period.

### 3.2. Virological Findings

The median age for confirmatory urine testing was 11 days (IQR 10–11; range 9–23). Urine viral loads ranged from <10^4^ to >10^7^ IU/mL. Plasma CMV DNA was tested in eight newborns, as plasma sampling was introduced after the initial phase of the pilot; all tested samples were positive, with a median viral load of 15,200 IU/mL (IQR 9185–35,400). There was notable variability in urine and plasma viral loads, but due to the small sample size, no correlation analysis with clinical severity was performed.

### 3.3. Clinical Findings and Antiviral Treatment

None of the confirmed cases were clinically suspected at birth, and following comprehensive postnatal evaluation, six of ten newborns (60%) met the protocol-defined criteria for symptomatic cCMV. In several of these infants, classification was driven primarily by abnormalities identified on neuroimaging performed as part of structured post-screening risk stratification rather than by clinically apparent disease at birth. The main imaging findings included lenticulostriate vasculopathy (LSV), white matter abnormalities, and germinolytic (mainly subependymal) cysts. For transparency, the individual clinical, virological, imaging, audiological, ophthalmological, and treatment characteristics are summarized in Table 1.

All ten confirmed cases passed newborn hearing screening at birth, and audiological assessment with AABR was normal in all cases. Ophthalmological examination was normal in all ten cases. Antiviral therapy was initiated in all six newborns classified as symptomatic, primarily because of central nervous system involvement. Treatment was started at a median age of 18 days of life (IQR 10–35 days). Oral valganciclovir was used in all treated infants. The four newborns classified as asymptomatic did not receive antiviral therapy.

The intended duration of antiviral treatment was six months. Five infants completed the planned course, whereas in one infant, treatment was discontinued after one week at parental request. No serious treatment-related adverse events were observed. Transient neutropenia occurred in two infants, but absolute neutrophil counts did not fall below 500 cells/mm^3^. In one infant, mildly elevated liver enzyme levels were documented on a single occasion and had normalized at the next follow-up assessment.

**Figure 1:**
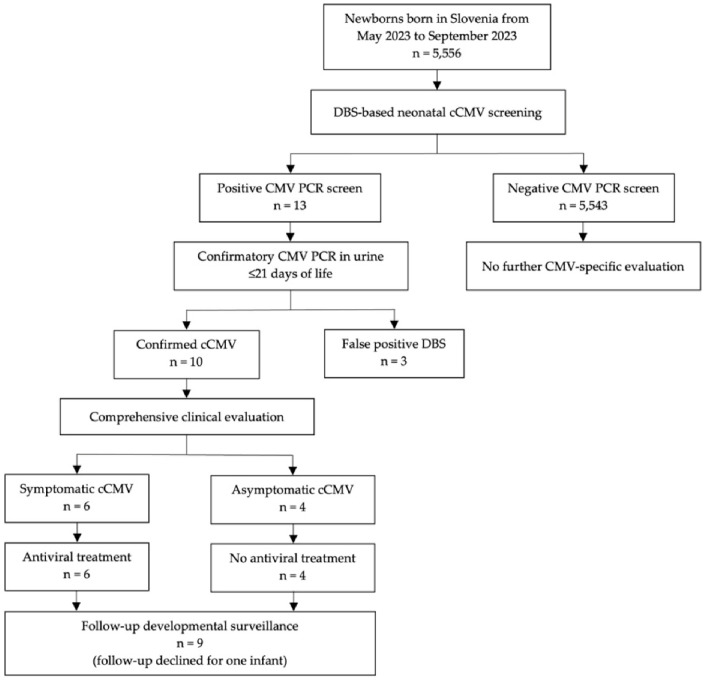
Outcome of universal newborn screening for congenital cytomegalovirus infection using dried blood spots. DBS: dried blood spot; CMV: congenital cytomegalovirus; cCMV: congenital cytomegalovirus infection; PCR: polymerase chain reaction.

All newborns with confirmed cCMV, whether symptomatic or asymptomatic, were enrolled in regular audiological and neurodevelopmental follow-up in accordance with the national protocol and international recommendations [4,9]. No screen-positive newborns were lost to confirmatory evaluation; however, in one case the parents declined further follow-up assessments. The overall outcome of universal DBS screening for cCMV is presented in Figure 1.

### 3.4. Comparison with Historical Registry-Based Clinical Detection

According to registry-based national data, 37 clinically identified cCMV cases were recorded in Slovenia between 2003 and 2022 among 395,223 live births, corresponding to a historical clinically ascertained detection rate of 0.09 per 1000 live births [23]. In the present pilot, 10 confirmed cases were identified among 5556 screened newborns, corresponding to a lower-bound birth prevalence of 1.80 per 1000 live births. This difference was statistically significant (*p* < 0.001) and illustrates substantial under-ascertainment under routine clinical detection pathways.

## 4. Discussion

In this national implementation pilot, universal DBS-based newborn screening for cCMV proved feasible, demonstrating that testing could be incorporated into the existing Slovenian newborn screening infrastructure using routine DBS samples. The program yielded substantially higher case detection than routine selective clinical identification. Universal DBS screening enabled identification of newborns who were clinically unsuspected at birth and who would likely have remained undiagnosed under symptom-based and hearing-targeted approaches [3,4]. These findings support the practical value of integrating cCMV testing into an established population-based screening system and show that such an approach can be implemented within routine national screening workflows [7,18,19,20].

The lower-bound prevalence of cCMV observed in our study was lower than the pooled global estimate reported in the literature but should be interpreted in the context of methodological and population differences. Global prevalence estimates include data from both high- and low-/middle-income countries, whereas studies from high-income settings generally report lower prevalence. In addition, blood-based screening methods have been associated with lower detection rates than saliva- or urine-based screening. Because confirmatory urine testing in our study was limited to DBS-positive newborns, false-negative DBS results cannot be excluded; therefore, our findings likely represent a lower-bound estimate of the true prevalence. Geographic variation, maternal CMV seroprevalence, and population characteristics may also contribute to differences in reported prevalence across studies.

A key finding of this pilot was the marked difference between the lower-bound birth prevalence identified through universal DBS screening and the historical registry-based clinically ascertained detection rate in Slovenia [23]. Registry-based ascertainment reflects cases detected through routine clinical pathways and therefore represents observed diagnostic yield rather than true birth prevalence. The substantially higher yield observed in the screened cohort suggests pronounced under-ascertainment under symptom-based and hearing-targeted strategies. At the same time, these two estimates are not methodologically equivalent, and the observed difference may also reflect temporal changes in clinical awareness, access to diagnostic testing, and reporting practices. The comparison should therefore be interpreted as evidence of likely under-recognition in routine practice rather than as a direct comparison of formally equivalent prevalence estimates.

None of the confirmed cases were clinically suspected at birth, and all passed newborn hearing screening, with otoacoustic emissions. Because BERA was temporarily unavailable during the study period, initial hearing assessment relied on AABR screening, which may have failed to detect some cases of hearing loss. However, the absence of hearing loss at the initial evaluation is not unexpected and is consistent with previous evidence showing that most infants with cCMV are asymptomatic at birth and that the onset of CMV-related SNHL is often delayed. Continued longitudinal audiological follow-up therefore remains essential to detect progressive or late-onset hearing loss. This also highlights the intrinsic limitations of symptom-based and hearing-targeted strategies. From a public health perspective, this supports the rationale for a population-based strategy capable of identifying affected infants before symptoms become apparent or sequelae emerge.

An important finding of our study was that all infants with confirmed cCMV were clinically asymptomatic at birth despite several being subsequently classified as symptomatic following comprehensive postnatal evaluation. Because neuroimaging was performed systematically as part of post-screening risk stratification, this classification was partly evaluation-dependent and may not be directly comparable with that reported in cohorts relying primarily on clinically apparent disease or targeted testing [9,24,25,26,27,28,29,30]. In most cases, classification was driven predominantly by abnormalities identified on neuroimaging rather than by clinically apparent disease at birth. Routine use of brain US and, where indicated, MRI likely increased detection of subclinical central nervous system abnormalities and may therefore have contributed to a higher symptomatic classification rate than in selective screening cohorts. We therefore consider it important to distinguish between clinically apparent symptomatic disease and imaging-defined central nervous system involvement when interpreting treatment decisions and comparing results across studies. However, not all neuroimaging abnormalities were automatically attributed to cCMV-related brain disease, as some findings, particularly LSV, are non-specific and may be observed in conditions other than cCMV infection. In our clinical practice, neuroimaging findings were interpreted in the context of the overall clinical presentation, laboratory results, and additional organ involvement, rather than being considered diagnostic of cCMV brain disease in isolation.

Consistent with national recommendations and international consensus guidance, antiviral treatment was initiated in infants who met predefined treatment criteria [4,9]. Universal screening made it possible to identify and evaluate affected infants within the therapeutic window, which is essential for consideration of antiviral treatment. No serious treatment-related adverse events were observed in this pilot. However, because the cohort was small and follow-up remains ongoing, no conclusions can yet be drawn regarding long-term treatment benefit. Neurodevelopmental and audiological assessments at two years of age have been completed and are currently being analyzed, while all children continue to undergo standardized follow-up until school entry according to the national protocol.

Our findings are broadly consistent with experience from other large-scale DBS-based screening programs. The Ontario program demonstrated that population-based DBS screening can be integrated into an established NBS infrastructure and can identify infants who would otherwise likely remain undiagnosed [18]. Statewide data from New York similarly confirmed the feasibility and acceptability of universal DBS-based screening in routine practice [19]. Taken together with our results, these studies suggest that the principal unresolved questions no longer relate primarily to laboratory implementation, but rather to the long-term clinical impact of universal screening, health economic consequences, the organization of coordinated multidisciplinary follow-up and care, and the optimal strategy for post-screening evaluation and management. At the same time, further evaluation of the diagnostic sensitivity of DBS-based screening across different laboratory methods and clinical settings remains an important area of ongoing research.

Universal screening may also increase identification of infants at risk of later audiological or neurodevelopmental sequelae by enabling earlier enrolment into structured follow-up. Based on the observed lower-bound birth prevalence (1.80 per 1000 live births) and published estimates indicating that 10–15% of initially asymptomatic infants develop sequelae [5], universal screening would correspond to approximately 0.17–0.27 at-risk infants per 1000 live births. In contrast, selective screening (0.09 per 1000 live births), combined with estimated 40–60% risk among symptomatic infants [5], corresponds to approximately 0.04–0.06 at-risk infants per 1000 live births. Under these scenario-based assumptions, universal screening could yield an approximately three- to seven-fold increase in identification of infants at risk of clinically relevant long-term impairment. However, any estimates of downstream clinical benefit based on this pilot should be regarded as hypothesis-generating rather than as observed outcome data. The present study was not designed to quantify long-term impairment, nor to determine whether earlier identification through DBS screening improves neurodevelopmental or audiological outcomes at the population level.

The timing of fetal infection remains a key determinant of disease severity, with first-trimester infection carrying the highest risk of central nervous system injury and permanent sequelae [9]. In Slovenia, stored maternal serum samples obtained during routine prenatal screening may, when available, allow retrospective serological assessment to estimate the timing of maternal CMV infection. Although this approach was not systematically evaluated in the present pilot study, it may provide additional information to support clinical interpretation and individualized counseling.

These considerations also have implications for counseling. Universal screening inevitably identifies infants who may never develop clinically significant sequelae. Structured assessment of infection timing, organ involvement, and standardized severity scoring may help contextualize individual risk and reduce uncertainty for families [31,32,33,34,35,36]. In this setting, screening supports both intensified surveillance of higher-risk infants and evidence-based reassurance for those at low predicted risk. In this setting, screening supports both intensified surveillance of higher-risk infants and evidence-based reassurance for those at low predicted risk; however, this stratification does not change the follow-up protocol itself, but rather reflects increased clinical vigilance and a higher index of suspicion for potential long-term sequelae in this subgroup.

Several limitations of this study should be acknowledged. Confirmatory urine testing was restricted to DBS-positive newborns, precluding formal estimation of diagnostic sensitivity and allowing the possibility of false-negative DBS results. An additional methodological consideration relates to the use of a single 3.2 mm DBS punch in the primary screening assay. Although some programs use multiple punches to improve sensitivity, our approach incorporated repeat testing of initially negative samples and triplicate testing of positive samples using independent punches, meaning that most samples were effectively analyzed more than once. This strategy was intended to mitigate sensitivity limitations while maintaining feasibility within a population-based screening framework.

The study included all newborns delivered in Slovenian maternity hospitals between May and September 2023. However, because the study period encompassed only part of the year, the cohort represented only a fraction of annual live births, and seasonal variation in congenital CMV prevalence cannot be excluded. Notably, all false-positive results occurred during the early phase of the pilot, when the laboratory workflow was still being established, and no false positives were observed after protocol stabilization, suggesting that these findings likely reflect transient implementation-related factors, including early analytical variability and possible PCR inhibition effects in DBS samples, rather than inherent assay limitations. The number of confirmed cases was small, limiting precision and precluding meaningful subgroup analyses. Newborn hearing status at initial evaluation was assessed using AABR screening rather than full diagnostic testing. BERA was not available at the time of initial evaluation due to temporary equipment unavailability. Although all infants with confirmed cCMV were subsequently enrolled in a standardized national follow-up program including BERA and/or ASSR testing, the absence of immediate diagnostic audiological assessment at birth may limit early characterization of hearing outcomes. Systematic neuroimaging was performed as part of the post-screening evaluation and likely increased the proportion of infants classified as symptomatic compared with cohorts relying primarily on clinically apparent disease. Consequently, direct comparisons of symptomatic disease rates across studies should be interpreted with caution. In addition, MRI was not performed in all infants; therefore, the extent of CNS involvement could not be fully characterized in some cases. Finally, long-term outcome data are not yet available, limiting assessment of the prognostic significance of imaging abnormalities detected through screening.

Despite these limitations, this pilot demonstrates that universal DBS screening for cCMV can be embedded in a national NBS program and can identify clinically unsuspected infants early enough to enable confirmatory diagnosis, structured risk stratification, and enrolment into follow-up. Formal cost-effectiveness modeling was beyond the scope of this pilot study but represents a necessary next step before consideration of national implementation.

## 5. Conclusions

In this national implementation pilot, universal DBS screening for cCMV was operationally feasible within the existing Slovenian NBS infrastructure and identified clinically unsuspected cases that would likely have remained undiagnosed under routine selective detection pathways. These findings support the practical value of integrating cCMV testing into an established population-based screening system. Although DBS-based screening does not eliminate all limitations related to assay sensitivity, its scalability, logistical simplicity, and compatibility with established NBS workflows make it an attractive population-level approach. Larger population-based studies with long-term outcome data and formal health economic evaluation are required to clarify clinical impact, cost-effectiveness, and optimal implementation pathways for universal DBS screening for cCMV.

## Figures and Tables

**Table 1:** Clinical, virological, imaging, audiological, ophthalmological and treatment characteristics of newborns with congenital cytomegalovirus infection.

Case	Sex	GA (Weeks)	Urine CMV (IU/mL)	Plasma CMV (IU/mL)	Clinical Findings	Brain US	Brain MRI	Abdominal US	AABR	Ophthalm. Exam	cCMV Classification	Antiviral Therapy	Therapy Adverse Effects	Follow-Up
1	female	39	>10^7^	N/A	none	normal	N/A	normal	normal	normal	asympt.	no	N/A	yes
2	male	40	qualitatively positive *	N/A	none	normal	N/A	normal	normal	normal	asympt.	no	N/A	yes
3	male	40	>10^7^	8960	none	LSV	N/A	normal	normal	normal	asympt.	no	N/A	yes
4	male	41	>10^7^	28,600	none	GC, LSV	WM injury	normal	normal	normal	sympt.	yes	neutropenia, elevated liver enzymes	yes
5	female	39	>10^7^	42,200	none	GC, LSV	normal	normal	normal	normal	sympt.	yes	no	yes
6	female	39	>10^7^	9410	none	LSV	N/A	splenomegaly	normal	normal	sympt.	yes	no	yes
7	female	39	>10^7^	18,200	SGA	normal	N/A	splenomegaly	normal	normal	asympt.	no	N/A	no ***
8	male	35	<10^4^	12,200	none	BG injury	normal	splenomegaly	normal	normal	sympt.	yes	no	yes
9	male	35	<10^4^	46,500	none	LSV, BG injury	WM injury	splenomegaly	normal	normal	sympt.	yes	neutropenia	yes
10	male	40	363,000	1970	none	LSV	WM injury	normal	normal	normal	sympt.	yes **	no	yes

* Quantitative value not available. ** Therapy discontinued after one week at parental request. *** Follow-up declined by parents. AABR: automated auditory brainstem response; asympt.: asymptomatic; BG: basal ganglia; CMV: congenital cytomegalovirus; cCMV: congenital cytomegalovirus infection; GA: gestational age; GC: germinolytic cyst; LSV: lenticulostriate vasculopathy; MRI: magnetic resonance imaging; N/A: not applicable; Ophthalm.: ophthalmological; SGA: small for gestational age; sympt.: symptomatic; US: ultrasonography; WM: white matter.

## Data Availability

The data presented in this study are available from the corresponding author upon reasonable request. The data are not publicly available due to privacy and ethical restrictions.

## Supplementary Materials

The following supporting information can be downloaded at: https://www.mdpi.com/article/10.3390/ijns12030048/s1, Management protocol for patients with congenital cytomegalovirus infection.

## Abbreviations

The following abbreviations are used in this manuscript:

AABR	Automated auditory brainstem response
ADC	Apparent diffusion coefficient
BG	Basal ganglia
CI	Confidence interval
cCMV	Congenital cytomegalovirus infection
CMV	Cytomegalovirus
Ct	Cycle threshold
DBS	Dried blood spot
DNA	Deoxyribonucleic acid
DWI	Diffusion-weighted imaging
GA	Gestational age
GC	Germinolytic cyst
IC	Internal control
IQR	Interquartile range
LSV	Lenticulostriate vasculopathy
MRI	Magnetic resonance imaging
N/A	Not applicable
NBS	Newborn screening
nHL	Normalized hearing level
PCR	Polymerase chain reaction
qPCR	Quantitative polymerase chain reaction
SGA	Small for gestational age
SNHL	Sensorineural hearing loss
SWI	Susceptibility-weighted imaging
US	Ultrasonography
WM	White matter
