# Supplementary material for: Universal Dried Blood Spot Screening for Congenital Cytomegalovirus: A Slovenian National Implementation Pilot
Source: Int J Neonatal Screen. 2026 Jun 29;12(3):48. doi: 10.3390/ijns12030048 (PMC13398074; doi:10.3390/ijns12030048)
Supplement: Supplementary file 1 [file IJNS-12-00048-s001.zip › IJNS-4281382-supplementary.pdf]

Management Protocol for Patients with Congenital Cytomegalovirus Infection

Age	Evaluation / Follow-up
At diagnosis confirmation	Clinical examination<br>Neurological examination<br>Hematological blood tests (complete blood count and differential white blood cell count)<br>Blood biochemistry tests (serum electrolytes, urea, creatinine, liver enzymes, total and direct bilirubin)<br>Ophthalmological examination<br>Hearing assessment (BERA and/or ASSR)<br>Imaging studies (cranial ultrasound and/or brain MRI, abdominal ultrasound)
1 month of age	Clinical examination<br>Neurological examination<br>Hematologic blood tests (complete blood count and differential white blood cell count)<br>Blood biochemistry tests (serum electrolytes, urea, creatinine, liver enzymes, total and direct bilirubin)<br>Referral to developmental follow-up clinic
3 months of age	Clinical examination<br>Neurological examination<br>Hematologic blood tests (complete blood count and differential white blood cell count)<br>Blood biochemistry tests (serum electrolytes, urea, creatinine, liver enzymes, total and direct bilirubin)<br>Cranial ultrasound (depending on previous imaging findings)<br>Hearing assessment (BERA and/or ASSR)
6 months of age	Clinical examination<br>Neurological examination<br>Hematologic blood tests (complete blood count and differential white blood cell count)<br>Blood biochemistry tests (serum electrolytes, urea, creatinine, liver enzymes, total and direct bilirubin)<br>Cranial ultrasound (depending on previous imaging findings)<br>Hearing assessment (BERA and/or ASSR)
12 months of age	Clinical examination<br>Neurological examination<br>Hearing and balance assessment (BERA and/or ASSR)<br>Ophthalmological examination (for symptomatic children)*<br>Psychological assessment (Bayley-III, ABAS-3)
18 months of age	Clinical examination<br>Neurological examination
24 months of age	Clinicalal examination<br>Neurological examination<br>Hearing and balance assessment (BERA and/or ASSR)<br>Speech and language therapy assessment<br>Psychological assessment (Bayley-III, ABAS-3, and CBCL)<br>Brain MRI
Annually until school entry	Clinical examination<br>Neurological examination<br>Hearing and balance assessment
Before school entry	Psychological assessment (WPPSI-III, ABAS-3, and CBCL)

ABAS-3 – Adaptive Behavior Assessment System, Third Edition

ASSR – Auditory Steady-State Responses

BERA – Brainstem-evoked response audiometry

CBCL – Child Behavior Checklist

MRI – magnetic resonance imaging

WPPSI-III – Wechsler Preschool and Primary Scale of Intelligence, Third Edition

* Further ophthalmologic follow-up is recommended in cases of ocular involvement
